# Cathepsin S: investigating an old player in lung disease pathogenesis, comorbidities, and potential therapeutics

**DOI:** 10.1186/s12931-020-01381-5

**Published:** 2020-05-12

**Authors:** Ryan Brown, Sridesh Nath, Alnardo Lora, Ghassan Samaha, Ziyad Elgamal, Ryan Kaiser, Clifford Taggart, Sinéad Weldon, Patrick Geraghty

**Affiliations:** 1 Airway Innate Immunity Research (AiiR) Group, Wellcome-Wolfson Institute for Experimental Medicine, School of Medicine, Dentistry and Biomedical Sciences, Queen’s University Belfast, Belfast, Northern Ireland UK; 2 Division of Pulmonary & Critical Care Medicine, Department of Medicine, State University of New York Downstate Medical Centre, Brooklyn, NY USA; 3 Department of Cell Biology, State University of New York Downstate Medical Centre, Brooklyn, NY USA

## Abstract

Dysregulated expression and activity of cathepsin S (CTSS), a lysosomal protease and a member of the cysteine cathepsin protease family, is linked to the pathogenesis of multiple diseases, including a number of conditions affecting the lungs. Extracellular CTSS has potent elastase activity and by processing cytokines and host defense proteins, it also plays a role in the regulation of inflammation. CTSS has also been linked to G-coupled protein receptor activation and possesses an important intracellular role in major histocompatibility complex class II antigen presentation. Modulated CTSS activity is also associated with pulmonary disease comorbidities, such as cancer, cardiovascular disease, and diabetes. CTSS is expressed in a wide variety of immune cells and is biologically active at neutral pH. Herein, we review the significance of CTSS signaling in pulmonary diseases and associated comorbidities. We also discuss CTSS as a plausible therapeutic target and describe recent and current clinical trials examining CTSS inhibition as a means for treatment.

## Proteases in pulmonary diseases

Research in the last 60 years has demonstrated that proteases are critical contributors to pulmonary disease pathophysiology. Initially known as protein-degrading enzymes with a restricted spectrum of substrates, recent studies have revealed that the diversity of protease substrates and biological effects triggered by their processing is vast [1, 2]. Proteases are primarily known for their matrix degradation capabilities, but also play significant roles in other biological mechanisms such as angiogenesis, growth factor bioavailability, cytokine processing, receptor shedding and activation, as well as cellular processes including migration, proliferation, invasion, and survival [3]. Importantly, protease activity requires tight regulation, and disruption of the close interplay between proteases, substrates and inhibitors may contribute to the pathogenesis and progression of a variety of pulmonary diseases, including muco-inflammatory diseases such as cystic fibrosis (CF) and chronic obstructive pulmonary disease (COPD), idiopathic pulmonary fibrosis (IPF), as well as infection [4]. In pulmonary diseases with a high neutrophil burden such as CF, a protease:antiprotease imbalance is frequently observed. The activity of proteases such as neutrophil elastase (NE) in the respiratory tract is regulated by antiproteases, such as α1-antitrypsin (A1AT) [5], secretory leukoprotease inhibitor (SLPI) [6] and elafin [7]. However, in diseases like CF, the antiproteases are overburdened by their cognate proteases and this imbalance can result in chronic airway inflammation, decreased mucociliary clearance, mucus obstruction, extracellular matrix (ECM) remodeling, increased susceptibility to infection and impaired immune responses [8]. Classically, NE was deemed the primary culprit in pulmonary disease pathogenesis, however, the contributions and importance of other proteases are now being recognized [9, 10]. There are many families of proteases, including metalloproteinases (matrix metalloproteinases, adamalysins, or pappalysins), serine proteases (elastase, coagulation factors, plasmin, tissue plasminogen activator, urokinase plasminogen activator), and the cysteine proteases (such as cathepsins). In this review, we will focus on one cysteine protease in particular, cathepsin S (CTSS), and outline the research supporting its growing importance in pulmonary diseases and the potential of targeting of CTSS as a therapeutic option.

## CTSS expression, production and function

CTSS plays a significant role in various intracellular and extracellular processes, including proteolysis [11] and major histocompatibility complex (MHC) class II-mediated immune responses [12]. CTSS is one of 15 cathepsin proteases encoded in the human genome that partake in various cellular processes [13–15]. They are categorized into three distinct protease subclasses determined by the enzyme’s active site catalytic residue; cysteine (B, C, F, H, K, L, O, S, V, W and X), aspartic (D and E), and serine (A and G) proteases [2]. CTSS is one of 11 cysteine cathepsin proteases, which is the largest cathepsin subclass. Cathepsins B, C, F, H, L, O, and X are expressed ubiquitously in human tissues and cells [16]. However, cathepsins K, W, V, and S are localized to certain tissues or cells [2]. CTSS is mainly found inside the lysosomal/endosomal compartments of antigen-presenting cells, such as B cells, macrophages, dendritic cells, but is also produced by epithelial cells, smooth muscle cells, endothelial cells, and neutrophils [17–21].

### CTSS production, activation, and secretion

The *Ctss* gene is found at the 1q21 chromosome in humans and, like all lysosomal cathepsins, is translated into a prepro-enzyme before being converted into a mature active state [22]. This acts as an important initial regulatory mechanism following the translation of the protein and during its localization to the lysosome [23]. Prepro-CTSS is composed of 331 amino acids [24] and contains three distinct domains; a signal domain, a propeptide domain, and a mature domain [22]. The secretion of CTSS usually occurs via vesicular exocytosis with elevated intracellular Ca^2+^ levels resulting in the fusion of the lysosome with the plasma membrane and the release of its contents into the extracellular space [25]. See the review by Wiederanders and colleagues for comprehensive discussion of cysteine cathepsin processing and proenzyme functions [26]. CTSS release is regulated by several factors including pro-inflammatory cytokines, such as IL-1β, TNF-α, IL-4 and IL-13 which have been shown to induce CTSS [27–30]. This may also be relevant in the context of inflammatory disease as CTSS is released from resident and recruited immune cells and inflamed tissue. CTSS has a reactive nucleophilic cysteine (Cys25) within its active site that is sensitive to hydrogen peroxide exposure, with the formation of sulfenic acid and converted to sulfinic acid [31]. This is partially reversed with antioxidant treatment [31]. Cysteine cathepsins require reducing and mildly acidic conditions for optimal activity and proteolytic removal of the propeptide domain in order to become active, which can be achieved autocatalytically or through the action of other proteases, which is facilitated at acidic pH as found in the endolysosomes [32, 33]. The autocatalytic processing of proform CTSS is regulated by negatively charged molecules such as glycosaminoglycans (GAGs) and polysaccharides including dextran sulfate and by changes in pH [34–36]. However, it is worth noting that this pH-dependent regulation of cathepsins may be less relevant when discussing CTSS as unlike other members of the lysosomal cathepsin family that require an acidic pH, CTSS has potent endoproteolytic activity at a broad pH range, which allows CTSS to be proteolytically active at neutral pH found in the healthy lung [23]. Interactions with ECM components such as GAGs may stabilize or alter cathepsin activity in the extracellular milieu at neutral pH. In addition, secreted CTSS can remain bound to the plasma membrane and has been detected in exosomes [37, 38]. Once secreted, the CTSS proenzyme has a half-life of 1-18 h depending on the origin of the cell [39]. These factors may help stabilize CTSS activity and protect against inactivation in the extracellular milieu, but may pose an additional hurdle to the development of efficacious CTSS inhibitors.

### CTSS functions

Intracellularly, CTSS is best known for its role in antigen presentation [40, 41]. Upon delivering an antigen into the endolysosomal pathway, the invariant chain (Ii) of MHC class II must be cleaved to form a class II-associated leupeptin-induced peptide (CLIP) fragment, thereby allowing subsequent exogenous antigen binding [41]. The proteolytic cleavage of Ii is catalyzed by active CTSS and other proteases [41]. The CLIP fragment is then cleaved, translocated to the plasma membrane of the antigen-presenting cell to ultimately activate CD4+ T cells [41, 42]. CTSS-mediated cleavage of li is not only important for presentation but also mediates dendritic cell motility [41, 42]. CTSS has also been found to be crucial in regulating toll-like receptor (TLR) 9 signaling. TLR9 recognizes unmethylated CpG DNA and translocates from the endoplasmic reticulum to endolysosomal compartments following stimulation where it can be cleaved at the N- or C-terminal by CTSS, producing fragments that inhibit (solubleTLR9) or act as a functional TLR ectodomain, respectively [43]. In addition, a role for CTSS in alum-induced IL-1β secretion was recently reported [44].

Extracellular cathepsins are involved in matrix remodeling via degradation of abundant structural components in the ECM, such as collagen and elastin [45]. CTSS has potent elastolytic and collagenolytic activities that serve an important role in several biological processes such as tissue remodeling. The ECM structure is organized and maintained through a balance of synthesis and degradation by proteolytic processing, including CTSS [45–47]. This processing may occur both extracellularly and intracellularly, with the degradation of ECM proteins internalized by endocytosis [13, 48]. CTSS also cleaves non-matrix proteins such as cytokines [49, 50], chemokines [51] and antimicrobial proteins (AMPs) [52], and can function as a sheddase releasing ectodomains of cell adhesion proteins and membrane receptors [53], thereby modulating their activity. Dysregulated CTSS activity and extracellular localization are associated with a number of pathological conditions that will be discussed within the following section of this review.

## Status and function of CTSS in pulmonary diseases

CTSS is particularly relevant in the context of pulmonary disease due to its ability to exert elastase activity, inactivate airway host defense proteins, induce ECM remodeling and alter mucus production across a wide pH range. Respiratory acidosis or alkalosis, characterized by altered partial pressure of CO_2_ in arterial blood due to altered alveolar ventilation and consequent insufficient elimination of CO_2_ from the blood, frequently occurs in several pulmonary diseases, such as bronchopneumonia (acidic), asthma (acidic), COPD (acidic), anemia (basic), pulmonary embolism (basic) and adult respiratory syndrome (basic). Therefore, altered pH is observed in diseases compared to the normal lung, which has a conservative range of pH similar to blood of 7.38-7.43 [54]. Equally, the pH of the exhaled airway vapor condensate is reported to vary from 6.85 to 7.65 [55]. Additionally, CTSS is expressed in numerous pulmonary cell types including alveolar macrophages, fibroblasts, and epithelial cells [56]. Within this section and in Table 1, we outline pulmonary diseases with altered CTSS levels, the role of CTSS as a biomarker or prognostic indicator, as well as findings from animal models demonstrating a significant role of CTSS in pulmonary diseases.

**Table 1:** The status and pathophysiological effects of CTSS in pulmonary and extra-pulmonary diseases

Pulmonary disease	CTSS signaling	Pathology	Potential therapeutics	References
COPD	Elevated in smokers and COPD patients Active in healthy lung at neutral pH, which could influence disease initiation	CTSS regulates lung inflammation and epithelial apoptosis CTSS levels corresponds to disease severity, i.e. FEV_1_/FVC and DLCO%	PP2A activators reduced CTSS expression and lung function decline in vivo	[57–59]
Asthma	CTSS genetic polymorphisms are linked to susceptibility of patients to asthma	CTSS-induced atopic dermatitis in mice PAR2 pathway results in CD4+ T cell differentiation, which is also involved in MHC class II expression	Methylprednisone reduced serum CTSS levels. CTSS inhibitor decreased inflammation and the number of eosinophils	[60–65]
PAH	Elevated CTSS in PAH patients and reduced elastic lamina and subsequent smooth muscle hypertrophy	Linked to atheroma formation and vascular remodeling in humans and rodent disease models.	Osthole modulated CTSS responses and reduced PAH in rats	[66–68]
Cystic Fibrosis	Negative correlation with lung function	Causes epithelial activation of the sodium channel, cleavage of surfactants, inactivation of βdefensins, and mucus production	CTSS inhibitor VBY-999 decreased inflammation, lung damage and mucous plugging in the lungs, partially via inhibition of PAR2	[69–73]
Cancer	High levels of CTSS expression in circulating tumor cells of SCLC CTSS-degraded Decorin	*Ctss*^−/−^ mice exhibit impaired micro-vessel growth during wound healing,	In vivo inhibition of CTSS decreased tumor growth in colorectal tumor models but unknown in lung cancer	[18, 74–78]
Sarcoidosis	Elevated CTSS levels in serum and histology samples	CTSS correlates with sarcoid diagnosis		[79]
IPF	High levels of CTSS expression	CTSS expression predicts disease progression		[80, 81]
**Extra-pulmonary diseases and outcomes**	**CTSS signaling**	**Pathology**	**Potential therapeutics**	**References**
Muscle function		*Ctss*^−/−^ mice have healthier skeletal muscle, reduced myofiber degeneration, fibrosis and improved running performance	Targeting of CTSS may improve muscle function	[82]
Sjögren’s syndrome	Elevated CTSS in patient tears	CTSS degrades tear proteins	CTSS inhibitor reduced symptoms in a mouse model	[83, 84]
Lupus		CTSS influences MHC class II-processing and T and B cell priming		[85]
Autoimmune encephalomyelitis		Double knockout of *Ctsb* and *Ctss* modulates MHC-II processing/presentation of myelin oligodendrocyte glycoprotein	Double knockout of *Ctsb* and *Ctss* protected mice from experimental autoimmune encephalomyelitis	[86]
Cardiovascular disease	CTSS is elevated in atheromas and in their surrounding tissues	*Ctss*^−/−^*ApoE*^−/−^ mice in a model of abdominal aortic aneurysm have reduced disease progression	CTSS inhibitor, RO5444101, decreased osteogenic activity, elastin degradation, plaque size, macrophage accumulation, growth differentiation factor-15, and calcification	[87–89]
Diabetes and obesity	CTSS levels reduce after gastric surgery	*Ctss* deficiency reduces blood glucose	Orally active small-molecule CTSS inhibitors reduced hepatic glucose production	[90, 91]
Pain	Elevated CTSS are observed in chronic colitis	CX3CL1/fractalkine is cleaved by CTSS resulting in the promoting of pain signaling. CTSS expression promotes itch, via PAR2/4-signaling		[92–94]

**Fig. 1:**
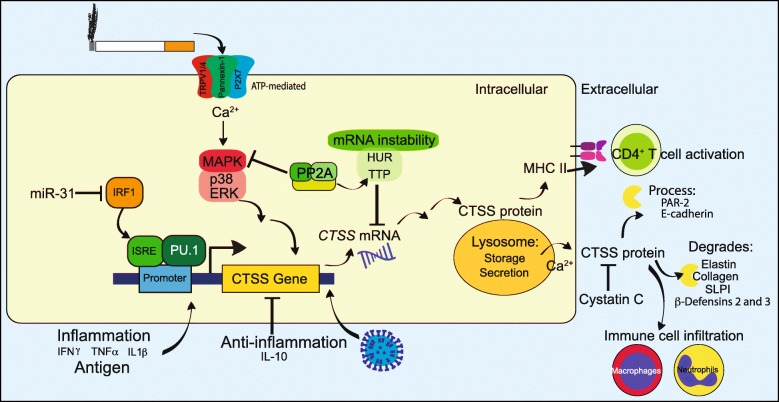
Mechanisms of CTSS regulation and function

### Chronic obstructive pulmonary disease (COPD)

COPD, principally caused by exposure to cigarette smoke and air pollution, is currently the third leading cause of death affecting 328 million worldwide and is expected to be the leading cause of death within the next 15 years [95]. Proteases are well known to play an important role in the pathogenesis of COPD [1, 9]. Elevated CTSS is observed in lung tissue, bronchoalveolar lavage fluid (BALF) and plasma of COPD patients as well as current smokers with or without COPD [57–59]. Importantly, elevated CTSS activity is observed in COPD [57] and appears to increase with disease severity [58]. As discussed previously, among the proteases associated with lung diseases, CTSS is unique in the sense that it has activity at neutral pH, i.e. it could be active within the lung and may contribute to disease initiation. This suggests that elevated CTSS could be one of the first insults in COPD development [54, 56]. Only 15–20% of smokers develop COPD, thereby suggesting that genetic, environmental or other as yet unknown factors contribute to the pathogenesis of COPD [96]. In addition, genetic polymorphisms of *Ctss* have been linked to a higher risk for COPD [97]. Therapeutic inhibition or genetic knockdown of CTSS reduced inflammation, epithelial apoptosis and lung damage in an IFNγ-induced model of emphysema [98]. A negative feedback loop may exist in COPD, where cysteine cathepsins degrade SLPI [69], a key pulmonary host defense protein and NE inhibitor, which in turn may interfere with the ability of SLPI to inhibit IFNγ-induced CTSS [57]. One of the most important environmental risk factors of COPD, cigarette smoking, increases the level of CTSS via multiple mechanisms including inhibition of protein phosphatase 2A (PP2A), which in turn increases CTSS expression and activity [99, 100]. Recently, we demonstrated that *Ctss* deficient mice were resistant to smoke-induced loss of lung function and that restoration of PP2A activation inhibited smoke-induced increases in CTSS expression and loss of lung function [99]. Interestingly, decreased PP2A was also detected in neutrophils from patients with A1AT deficiency, which is associated with COPD pathogenesis [100]. Cigarette smoke also activates the P2X7 receptor in bronchial epithelial cells, which leads to higher CTSS levels [58]. Elevated CTSS levels in BALF correspond to disease severity based on the GOLD staging of COPD in active smokers, and negatively correlate with FEV_1_/FVC and DLCO% [58, 101]. It remains to be determined whether CTSS plays a role in e-cigarette associated pulmonary inflammation and damage [102–104]. Breath condensate pH is lower in patients with COPD and bronchiectasis compared to control subjects [105], which could further influence CTSS activity. Recently, Wartenberg and colleagues demonstrated that pro-CTSS is resistant to cigarette smoke extract inhibition of autocatalytic maturation despite an increase of carbonylation [31]; thereby maintaining its activity in the presence of smoke exposure. However, CTSS activity is sensitive to oxidative stress and other components of cigarette smoke [31]. Overall, these recent studies demonstrate that CTSS may be an important factor contributing to COPD pathogenesis and severity.

### Asthma

Asthma, one of the most common chronic non-communicable diseases, has complex genetic and environmental interactions with allergic sensitivity, obesity and cigarette smoking playing a role in its pathogenesis. Corticosteroids are one of the first-line agents for asthma control and for treatment of asthma exacerbation, and response to corticosteroids has a role in prognostic prediction [60]. Asthma patients treated with methylprednisone had lower levels of serum CTSS protein [61]. Similar to COPD, *Ctss* polymorphisms have also been linked to the susceptibility of patients to asthma [62, 63]. Findings from animal models have also linked CTSS expression with asthma pathogenesis and atopy. Atopic asthma is seen in approximately 50% of patients with asthma and atopic sensitization may be important in the pathogenesis of severe asthma specifically in childhood onset asthma [106, 107]. CTSS is upregulated in models of eosinophilic inflammation and airway hyperreactivity; IL-13 overexpressing mice, ovalbumin (OVA)-induced allergy model, and mice sensitized with *Aspergillus fumigatus* antigen [108, 109]. Knockout of *Ctss* or prophylactic administration of a CTSS inhibitor resulted in decreased airway inflammation and BALF eosinophil counts compared to controls in OVA challenged mice [64]. In addition, prophylactic CTSS inhibitor treatment reduced OVA-induced mucus obstruction in the airways [64] suggesting a possible therapeutic role for prophylactic CTSS inhibitor treatment in patients with asthma. These findings may be secondary to alterations in MHC class II–Ii processing and subsequent peptide loading [110]. CTSS induced itch in human skin and atopic dermatitis in mice via activation of protease-activated receptor (PAR)-2 and − 4 [65, 92]. In mice, activation of PAR2 induced dendritic cell maturation and subsequent CD4^+^ T cell differentiation leading to increased dermal inflammation and chronic scratching behavior [65, 92]. Finally, the pH of exhaled airway vapor condensate is significantly lower in patients with acute asthma (pH 5.23) than in control subjects (pH 7.65) [55]. This favors the proteolytic activities of cathepsins, including CTSS. Therefore, CTSS may be linked to inflammation, atopy and susceptibility to asthma and dermatitis.

### Pulmonary arterial hypertension (PAH)

Pulmonary arterial hypertension (PAH) is a progressive disease of the lung vascular system characterised by vascular remodelling and narrowing of the blood vessels, resulting in increased vascular resistance and right heart failure [111]. PAH can be idiopathic, genetic or associated with drug and toxin exposures or medical conditions such as COPD [112] and CF [113]. Dysfunctional acid-base transport function is suggested to induce clinically relevant changes in blood pressure and could contribute to hypertension development [114]. Increased CTSS and degradation of elastic laminae were observed in the lungs of patients with idiopathic PAH and elevated CTSS activity in pulmonary arterial smooth muscle cells (PASMC) from the monocrotaline (MCT)-induced PAH rat model [115]. Administration of a CTSS inhibitor reduced MCT-induced CTSS levels and activity with concomitant preservation of the integrity of elastic laminae of the pulmonary arteries [115]. Inhibiting CTSS reduced PASMC proliferation, migration and reactive oxygen species (ROS) production, which are among the earliest pathobiological features of vascular remodelling, suggesting that CTSS plays a role in the pathogenesis of PAH [66, 67, 115]. In agreement with previous work, peroxisome proliferator-activated receptor (PPAR)-γ was identified as an important regulator of CTSS expression in SMCs [115, 116]. Yao et al. reported that osthole, a novel hypotensive agent purified from the Chinese medical plan *Angelica pubescens* Maxim, down-regulated MCT-induced CTSS levels while reducing PAH [117].

### Cystic fibrosis (CF)

CF is a genetic disorder resulting from loss of expression or function of the cystic fibrosis transmembrane conductance regulator chloride channel [118]. CF is a multi-organ disease; however, lung disease is the primary cause of morbidity and mortality in CF patients. Pulmonary manifestations include glandular hyperplasia as well as accumulation of viscous, neutrophil-dominated, mucopurulent debris in the airways which leads to chronic inflammation, recurring infections and lung remodeling as a sequela of altered host immune responses and a protease:antiprotease imbalance [119, 120]. The first reports of CTSS in the lungs of patients with CF linked CTSS activity to degradation of host AMPs βdefensins and lactoferrin [69, 121]. Subsequent work reported increased CTSS expression and activity as a feature of the CF lung in stable patients with CF and also in pediatric patient cohorts, with higher concentrations detectable in lower airways compared to upper airways [70, 122, 123]. Research shows that CTSS plays multiple roles in the pathophysiology of CF including activation of the epithelial sodium channel (ENaC) [71] and cleavage of host defense proteins including surfactants and LL-37 [69, 72, 121, 124]. One of the mechanisms that leads to elevated CTSS levels in CF is the alteration of miRNA-31 expression in bronchial epithelial cells, resulting in elevated CTSS levels via the miR-31/IRF-1/CTSS pathway [70]. Also, increased levels of CTSS measured in BALF from pediatric patients with CF negatively correlated with lung function suggesting that CTSS levels could be used as a possible prognostic biomarker or even a measure of progression of the disease [70]. In a recent study using the βENaC-transgenic mouse model of CF lung disease, pharmacological or genetic targeting of CTSS decreased inflammation and mucus plugging in the lungs mediated, at least in part, via inhibition of PAR2 suggesting a potential novel therapeutic avenue for CF [73]. Acidic pH levels are reported on the apical surface liquid of epithelial cells isolated from CF subjects compared to controls [125]. CTSS is also observed on ciliated airway cells and could possibly influence ciliary function [56] but further studies are required. Overall, this research suggests that CTSS could be a significant player in CF lung disease pathogenesis and progression.

### Lung Cancer

Given its ability to remodel the ECM, it is not surprising that there is a vast amount of research on the role of CTSS in cancer [126], however, data specific to lung cancer is relatively limited. Acidic extracellular pH plays an important role in the cancer microenvironment and contributes to cell invasion and metastasis potential [127]. Serum CTSS is associated with increased cancer mortality [128], and elevated protease expression, including CTSS, is associated with poor prognosis in numerous tumor types [129]. The loci that the *CTSS* gene is located on chromosome 1q21.2, which is amplified in many cancers [130] and this amplification could contribute to elevated CTSS levels observed in clinical cancer samples, in addition to infiltrating CTSS-rich immune cells. Decorin, a proteoglycan of the interstitial matrix, is degraded by CTSS in lung cancer and may be associated with lung cancer pathology [74]. CTSS proteolytic degradation of nidogen-1, an essential component of the basement membrane and a substrate of CTSS, is strongly associated with non-small cell lung cancer [131]. A recent study showed an increased and exclusive secretion of CTSS in circulating tumor cells of small cell lung cancer, which may predict distant metastasis and targeting CTSS may limit tumor dissemination to distal sites [132]. Interestingly, CTSS has also been linked to breast-to-brain metastasis via processing of the junctional adhesion molecule-B and subsequent blood-brain barrier transmigration [75].

Recently, CTSS was shown to play an important role in mediating Ca^2+^ homeostasis by regulating stromal interaction molecule (STIM) 1 trafficking, and inhibition of CTSS resulted in decreased cell migration and invasion [133]. In addition, CTSS promoted pericellular hydrolysis of the ECM within the tumor microenvironment, facilitating endothelial invasion [76]. CTSS is expressed in endothelial cells and may play an important role in angiogenesis; *Ctss*^−/−^ mice exhibit impaired microvessel growth during wound healing [18], and CTSS modulated angiogenic peptides involved in microvascular growth [77, 78]. There is an increasing recognition of the overlapping prevalence of COPD with lung cancer with the presence of COPD increasing the incidence of lung cancer and lung cancer mortality [134]. Further work is needed to delineate the role of CTSS in lung cancer and whether there is an association between elevated CTSS in COPD and lung cancer initiation and progression. PP2A activity may represent one such link. As previously mentioned, cigarette smoke increased the level of CTSS via inhibition PP2A, which may function as a tumor suppressor [99, 135]. Furthermore, cigarette smoke induced the expression of the oncoprotein CIP2A (cancerous inhibitor of PP2A), which down-regulates PP2A activity and enhances proteolytic responses [136], potentially creating a favorable inflammatory micro-environment that may promote tumor formation.

### Other pulmonary diseases

CTSS is also linked to several other pulmonary diseases, including sarcoidosis and IPF. Sarcoidosis a multisystem disease with pulmonary manifestations and recent research has shown that CTSS levels in serum as well as CTSS staining in histology specimens not only correlates with the diagnosis but also with the severity and steroid responsiveness suggesting the potential use of CTSS as a biomarker [79]. Similarly, levels of CTSS have been shown to predict disease progression in patients with IPF [80]. IPF is strongly associated with gastroesophageal reflux disease [137], a condition where acid flow could modulate lung pH and potentially enhance cathepsin activity. CTSS-degraded decorin is also being investigated as a potential biomarker for fibrotic lung disorders including IPF [81] in addition to lung cancer [74]. Interestingly, the endogenous cathepsin inhibitor, cystatin C, is a possible biomarker for IPF [138]. In a mouse model of bronchopulmonary dysplasia, genetic knockdown of *Ctss* protected against hyperoxia-induced lung injury [139]. *Ctss*^−/−^ mice displayed improved alveolarization, attenuated macrophage recruitment and fibroproliferative changes, and were protected from growth restriction and alveolar–capillary leak [139].

## Extra-pulmonary role of CTSS

This review focuses on the pulmonary role of CTSS. However, many lung diseases are associated with a high burden of comorbidities [112, 113, 140, 141]. CTSS is expressed by immune cells, smooth muscle cells, epithelial and endothelial cells suggesting that elevated levels of CTSS can circulate in the blood and thus have multiple effects beyond the lungs. Therefore, it is important to briefly discuss the potential impact of CTSS in non-pulmonary disorders often associated with chronic inflammatory lung disease (Table 1). Dysregulated CTSS expression or activity has been linked to several non-pulmonary diseases or outcomes of disease that are frequently observed as pulmonary comorbidities or additional symptoms such as cardiovascular disease and metabolic complications. Here we provide a brief overview of several pieces of data which suggest that CTSS may play a major role in extra-pulmonary disease progression and provide evidence that targeting CTSS may also be a plausible means to treat comorbidities associated with pulmonary diseases.

### Skeletal muscle dysfunction

Muscle fatigue and wasting is frequently observed in pulmonary diseases leading to a decline in pulmonary function, hypoventilation, inefficient cough, hypercapnia, and sleep disorders. CTSS expression is linked to muscle function with *Ctss*^−/−^ mice having healthier skeletal muscle with reduced myofiber degeneration and fibrosis, and improved running performance in a genetic model of muscular dystrophy [82]. These changes were linked with membrane adhesion complex stability and changes in utrophin, β-dystroglycan, and integrins. In addition, a muscular dystrophy-like phenotype is induced in skeletal muscle-specific transgenic *Ctss* overexpressing mice [82]. Hence, increased CTSS expression and activity may play a role in skeletal muscle pathology.

### Autoimmune diseases

Since CTTS responses are triggered by inflammatory stimuli and CTSS deficiency can attenuate MHC class II presentation, many investigators have focused on CTSS as a target in immunological disorders, such as lupus, rheumatoid arthritis, Sjögren’s syndrome and encephalomyelitis. CTSS promotes systemic lupus erythematosus by regulating MHC class II-mediated T and B cell priming, germinal center formation and B cell maturation into plasma cells [85]. Inhibition of CTSS in these models could reduce lupus progression [85] and autoimmune-triggered inflammatory responses in macrophages [142]. Double knockout of *Ctsb* and *Ctss* modulates MHC-II processing and presentation of myelin oligodendrocyte glycoprotein, and protects mice from experimental autoimmune encephalomyelitis [86]. Recently, CTSS activity was observed to be elevated in Sjögren’s syndrome patient tears, which results in tear protein degradation [83]. Systemic or topical administration of a CTSS inhibitor reduced symptoms in a mouse model of Sjögren’s syndrome [84]. Therefore, CTSS appears to play a role in autoimmune responses and represents a potential therapeutic target to treat these diseases in addition to the pulmonary diseases already outlined in this review.

### Cardiovascular disease

In a community-based cohort of elderly men, serum CTSS was associated with increased cardiovascular mortality [128], and CTSS was one of the first cathepsins observed in human atherosclerotic lesions [87]. CTSS is an important player in the pathogenesis of several cardiovascular diseases, such as atherosclerosis, post-myocardial infarction remodeling and abdominal aortic aneurysm (AAA) [143–145]. CTSS expression and activity are elevated in atheromas and surrounding tissue, possibly due to the presence of infiltrating immune cells [87], and colocalized with regions of elastin degradation in human coronary plaques [146]. *Ctss* deficiency reduced plaque formation and disease progression in atherogenic low-density lipoprotein receptor (*Ldlr*^−/−^) mice and apolipoprotein E (*ApoE*^−/−^) knockout mice [146, 147]. Leukocyte *Ctss* deficiency resulted in smaller plaque necrotic cores, reduced apoptosis, and decreased SMC content and collagen deposition and therefore may be a key player in plaque stability [148]. Elevated CTSS expression was observed in samples from patients with AAA [149], and knockdown of *Ctss* reduced disease progression in a mouse model of AAA [88]. The selective CTSS inhibitor, RO5444101, decreased osteogenic activity, elastin degradation, plaque size, macrophage accumulation, growth differentiation factor-15, and calcification in a mouse chronic renal disease model [89]. This is important as chronic renal disease accelerates the development of atherosclerosis. Recently, CTSS was also observed to play a significant role in injury-induced arterial damage in an animal model of stress-related neointimal hyperplasia [150].

### Diabetes

CTSS may be involved in the early dysregulation of glucose and insulin metabolism as higher serum CTSS was associated with decreased insulin sensitivity and an increased risk for developing diabetes [151]. Several studies have demonstrated a link between CTSS, diabetes and obesity. *Ctss* deficiency results in a robust reduction in blood glucose, even in diet-induced obesity animal models and aged mice [90]. Interestingly, suppression of CTSS signaling reduced hepatic glucose production without improving insulin sensitivity [90]. Diabetic rats have elevated CTSS, which was reversed with atorvastatin treatment [152]. Elevated circulating CTSS concentrations are associated with metabolic syndrome in overweight and obese adults [153]. Lifestyle changes in moderately overweight subjects resulted in reduced plasma CTSS levels [154]. This is possibly due to elevated levels of CTSS observed in adipose tissue and serum of obese patients. CTSS was identified as an important regulator of PAR2 mediated endothelial dysfunction that promotes microvascular diabetes complications [155].

### Other outcomes

Chronic pain is common in patients with chronic lung disease [156, 157] and CTSS activity has been linked to pain in several conditions. The inflammatory chemokine CX3CL1/fractalkine, associated with inflammatory lung diseases [158], is proteolytic cleaved by CTSS resulting in the promotion of pain signaling [50, 159]. Administration of recombinant CTSS into the colonic lumen produces significant nociceptive pain, similar to the pain observed in chronic colitis [160] and elevated levels of CTSS are observed in chronic colitis [93]. Importantly, recent data suggests that circulating CTSS protein levels are associated with increased mortality risk in elderly men and women [128]. Furthermore, CTSS participates in inflammatory processes associated with aging and neurodegeneration [161]. Therefore, CTSS is likely to have a role in aging associated diseases, such as COPD. Therefore, mounting data suggests that CTSS is a significant player in pulmonary and non-pulmonary disease progression that may impact on patient mortality. Research suggest that modulation of CTSS could be beneficial in a range of pulmonary diseases as well as extra-pulmonary comorbidities.

## Regulation of CTSS

Whilst CTSS has several important functions within the healthy cell, it is clear that in various pathological states as highlighted in this review, its dysregulation can lead to uncontrolled and damaging proteolytic activity. Therefore, in the physiological state, it is critical that CTSS responses are tightly regulated. This regulation is carried out at multiple levels including expression, zymogen activation, compartmentalization to lysosomes, secretion, and activity [13, 161]. Various mechanisms are employed at each of these regulatory levels to maintain cellular homeostasis. Within this section, we will discuss the mechanisms involved at each of these levels and highlight factors that can alter this regulation in the diseased state.

### Regulation of expression

CTSS is regulated at the expression level, both during transcription and post-transcriptionally. Under pro-inflammatory conditions, expression of *Ctss* is induced as a result of signaling by cytokines, most notably by IFNγ [162]. The *Ctss* promotor contains a functional IFN-stimulated response element (ISRE) to which the transcription factor IRF-1 binds, inducing CTSS transcription [163]. The expression of CTSS via IRF-1 has also been shown to be regulated by dysregulated miR-31 in CF bronchial epithelial cells [70]. Other transcription factors including PU.1 and PPARγ may also regulate CTSS expression [115, 164]. Additionally, inflammatory mediators including IL-1β and TNF-α have been shown to induce CTSS expression, whilst the anti-inflammatory cytokine IL-10 inhibits CTSS expression [165]. In addition to these inflammatory mediators, other molecules are also involved in the regulation of CTSS expression. As mentioned previously, PP2A negatively regulates CTSS expression in a COPD model of smoke-induced lung injury and in human bronchial epithelial cells and monocyte-derived macrophages [99]. This is highly relevant as PP2A is dysregulated in several inflammatory conditions including COPD, cancer, neurodegenerative and heart disease [166–168]. This may contribute to the upregulation of CTSS in these conditions and provide a possible new therapeutic target for treatment. Interestingly, CTSS expression is also regulated by proteins involved in the maintenance of circadian rhythms. Diurnal regulation of CTSS gene regulation has been observed following the rhythmic expression of the clock genes Period (Per) 1 and Per 2 [169]. This circadian regulation may be disrupted in asthmatics, suggesting a possible role for the dysregulation of circadian rhythms in chronic lung disease [170].

Post-transcriptional regulation of CTSS mRNA includes regulation by the stabilizing RNA-binding proteins human antigen R (HuR) and tristetraprolin (TTP). HuR binds to the 3′ UTR of the CTSS transcript thereby promoting stability of CTSS mRNA in endothelial cells and may contribute to the elevated CTSS levels seen in human atherosclerotic vascular diseases [171]. The availability of HuR binding sites is increased following adenosine-to-inosine RNA editing, a process that is promoted both by inflammatory stimuli [172] and hypoxia [173], providing an additional mechanism for the regulation of CTSS by both of these stimuli. TTP also regulates pro-inflammatory responses through the destabilization of mRNA and has a significant anti-inflammatory effect in the airways [174, 175]. PP2A, a previously discussed regulator of CTSS expression, enhances TTP destabilization of mRNA providing an additional mechanism for PP2A-associated regulation of CTSS [176]. Importantly, TTP expression is dysregulated in several diseases [177], which could enhance CTSS levels. Post-transcriptional regulation of CTSS expression by intracellular pathogens has also been demonstrated. Mycobacterium species including *M. tuberculosis* and *M. bovis* regulate CTSS to promote their intracellular survival. This regulation is mediated, in part, by miRNAs. Specifically, *M. bovis* induces the upregulation of miR-106b-5p, a miRNA that targets CTSS, in infected cells [178]. Additionally, IL-10 dependent inhibition of CTSS by *M. bovis* has been observed [179]. Regulation of CTSS provides a mechanism by which Mycobacterium can attenuate MHC class II surface expression and promote immune evasion [180, 181]. Nitrated fatty acids can downregulated CTSS, via S-alkylation of the Cys25 site [182]. Overall, it appears that regulation of the expression and production of CTSS is regulated by several mechanism, which appear to be cell and tissue specific.

### Regulation of activity

The regulation of cysteine cathepsin activity is carried out by their endogenous inhibitors, cystatins, thyropins, and serpins. Cystatin C is the most potent endogenous inhibitor of CTSS activity. Cystatin C is a selective protein inhibitor that is constitutively expressed by most cells, it contains a signal peptide that is responsible for its secretion through the cell membrane and as such is also found at high concentrations in extracellular fluids [183]. Cystatin C inhibits CTSS by physically blocking the active site preventing the binding of its substrate. The high extracellular concentrations of cystatin C allow it to act as an emergency inhibitor to neutralize excessive extracellular proteolytic activity [45, 184]. In addition to cystatin C, other members of the cystatin family also regulate CTSS including cystatin F [185]. These endogenous inhibitors provide an important regulatory mechanism for CTSS and this is highlighted by the fact that abnormal formation of cystatin C, which is a condition known as hereditary cystatin-C amyloid angiopathy, results in cerebral hemorrhage with amyloidosis [186]. The CTSS:cystatin C ratio is also increased in the plasma of COPD patients compared to healthy controls highlighting the importance of maintaining a protease:antiprotease balance in which the antiprotease levels exceed that of the protease [59].

In addition to endogenous inhibitors, other factors contribute to the regulation of CTSS activity. An important requirement for optimal CTSS activity is for the enzyme to be maintained in a reduced state as oxidation of the active site cysteine renders the enzyme inactive [187]. Therefore, reducing agents such as γ-Interferon-inducible lysosomal thiol reductase (GILT) are required to maintain CTSS in its reduced active state within the highly acidic, and occasionally oxidative, environment in the late endosomal/lysosomal compartment [188]. This regulation may extend beyond the lysosome with the observed secretion of GILT from activated macrophages, which may promote extracellular CTSS activity during inflammation [28]. However, a separate study demonstrated that GILT shortened the half-life of CTSS, resulting in decreased CTSS steady-state protein levels [189]. This suggests that GILT may regulate CTSS activity by reducing the amount of time the enzyme is active. Conversely, the phagosomal NADPH oxidase NOX2, an oxidizing agent, is an effective inhibitor of CTSS activity, possibly acting as a negative feedback regulatory system to prevent excessive protease activity during inflammation [190]. In contrast, ROS promotes the formation of cystatin C homodimers thereby leading to dissociation of CTSS and cystatin C and a loss of its inhibitory potential. Indeed, increases in ROS in catalase-silenced cells lead to increased CTSS activity [191], whereas maintenance of low intracellular ROS by symbiotic commensals including *Bacteroides vulgatus* promotes the ability of cystatin C to inhibit CTSS [192]. These opposing effects of reducing and oxidizing agents on CTSS and its endogenous inhibitor cystatin C highlight the complex interplay involved in maintaining proteolytic homeostasis. Interestingly, CTSS is less susceptible to oxidative stress induced by H_2_O_2_ in acidic pH conditions compared to other cysteine cathepsins [193]. The microbiome has also been shown to directly regulate CTSS activity via the production of small molecule dipeptide aldehydes that target the catalytic cysteines, blocking the active site of CTSS and preventing binding of substrates [194]. As previously discussed, CTSS plays a major role in antigen presentation and loading of the MHC class II molecule. This process is regulated by p41, a fragment of the MHC class II-associated Ii. p41 had initially been observed to form a complex with CTSL, acting as a competitive inhibitor by reducing its proteolytic activity, and was considered a specific inhibitor of CTSL [195]. More recent studies have demonstrated a weaker but still physiologically relevant inhibition of CTSS activity by the p41 fragment by the same mechanism [196].

CTSS has extracellular and intracellular functions that can influence physiological changes within the lungs and most importantly influence disease pathology (see Fig. 1 for known CTSS signaling). Targeting CTSS represents a potential therapeutic for several pulmonary diseases and their comorbidities. In addition, a number of upstream factors that modulate CTSS expression and/or production have been identified in various cells, tissues and models of disease. However, whether to target CTSS extracellularly or intracellularly requires further investigation and may be the key to unlocking the development of a safe and efficient anti-CTSS therapeutic for patients.

## CTSS therapeutic potential

Due to the involvement of CTSS in the pathogenesis of numerous diseases as highlighted in this review, there has been increasing interest in the inhibition of CTSS as a potential therapeutic target. Clinical trials to date have primarily focused on autoimmune diseases, including psoriasis, Sjogren’s syndrome and rheumatoid arthritis. We will discuss currently available CTSS inhibitors and clinical trials undertaken with these therapeutics to date. Additionally, we will examine the potential adverse effects of long-term CTSS inhibition.

### Therapeutic inhibition of CTSS

Several approaches have been utilized as CTSS inhibitors; these include small molecule inhibitors, neutralizing antibodies, recombinant proteins, and modulation of upstream CTSS regulators. Numerous preclinical studies have demonstrated beneficial effects in various disease models through the use of small-molecule inhibitors to target CTSS [48, 64, 73, 197, 198]. Small-molecule inhibitors are competitive inhibitors that block the enzyme’s active site preventing substrate binding. These small molecules are selected from large libraries of natural and synthetic compounds, typically with structures similar to known protease substrates, and screened for their inhibitory activity against CTSS [199]. To date, clinical trials have mostly relied on the use of these small molecule inhibitors to target CTSS due to their high selectivity and oral bioavailability [200]. In addition to these small molecule inhibitors, other therapeutic agents could be utilized to target CTSS expression and activity. Neutralizing antibodies raised against CTSS are a promising therapeutic option. Preclinical studies of the anti-CTSS antibody Fsn0503 have shown potential benefits, demonstrated by in vitro and in vivo models of colorectal carcinoma [76, 201, 202]. However, no clinical studies have yet been undertaken that include anti-CTSS antibodies. Additionally, recombinant CTSS propeptide has been used as a potent inhibitor of CTSS [203]. Both these antibody-based and protein-based therapies specifically target secreted CTSS. This could potentially help negate adverse effects associated with intracellular CTSS inhibition, namely, the blocking of MHC class II loading and subsequent cell-mediated immune responses [85, 142]. As an alternative to the direct targeting of CTSS, therapeutic agents that target regulators of CTSS expression or activity, as discussed in the previous sections, may have therapeutic potential. Increased production or instillation of the endogenous CTSS inhibitor cystatin C is an example of this alternative therapeutic. Indeed, preclinical studies have shown beneficial effects of the overexpression of cystatin C in a model of fibrosarcoma lung metastasis [204, 205]. While recombinant cystatin C therapy has not yet been investigated, treatment with recombinant cystatin from *Schistosoma japonicum* improved outcomes in 2,4,6-Trinitrobenzenesulfonic acid-induced murine model of colitis [206], highlighting the potential for similar therapies involving recombinant cystatin C. Alternatively, the targeting of other CTSS regulators including PP2A, IFNγ or IL-6 has shown positive outcomes in initial preclinical studies [207–210].

### Clinical trials to date

Many phase I and II clinical trials were undertaken to assess the safety, dosing, and efficacy of CTSS inhibitors in a variety of diseases [211–223]. Of these 13 studies, six have studied safety and tolerability in healthy volunteers and seven have examined efficacy in psoriasis, rheumatoid arthritis, coeliac disease, and Sjogren’s syndrome. These studies have all used small molecule inhibitors of CTSS, namely RO5459072, LY3000328, RWJ-445380, VBY-036 and VBY-891, all potent, selective and orally bioavailable compounds [224, 225]. These molecules have demonstrated good safety profiles but variable efficacy in improving disease outcomes. Initial trials launched by Celera in 2005 using the inhibitor CRA-028129 were discontinued following phase I clinical trials [226]. This was followed by phase IIa trials in rheumatoid arthritis and psoriasis with RWJ-445380, which were discontinued due to poor efficacy. RO5459072 passed a phase I trial in patients with coeliac disease, however, subsequent trials in patients with Sjogren’s syndrome showed no change in primary outcome measures. To date, there are no CTSS inhibitors that have become clinically available, primarily due to poor efficacy during phase II clinical trials. However, a phase II trial is currently underway with RO5459072 in psoriasis patients [124]. Given the remit of this review, it would be expected to address clinical trials undertaken to examine the efficacy of CTSS inhibitors in pulmonary disease, however, no such trials have been carried out despite promising preclinical data. Interestingly, a previous phase I dose-escalation study with the CTSS inhibitor LY3000328 noted that while there was an initial decrease in CTSS activity this is followed by a more prolonged period of increased CTSS concentration [225]. This effect was also observed following in vitro inhibition with the broad spectrum cathepsin inhibitor E-64 and recombinant cystatin C [227]. This highlights a potential feedback loop that maintains CTSS activity by stabilizing the CTSS protein following inhibition but we must be mindful of possible risks when utilizing broad range CTS inhibitors such as E-64 and recombinant cystatin C, such as possible modulation of MHCII processing and antigen presentation. Whether this has contributed to poor efficacy in phase II trials of CTSS inhibitors is unclear, however, a better understanding of these feedback mechanisms will be important to progress development of effective CTSS inhibitor-based therapies.

### Potential adverse effects

Clinical trials to date have shown that CTSS inhibitors are, in general, well-tolerated by patients and healthy volunteers. However, several minor adverse events have been noted including, nausea, headache, rash, pruritus, urticaria and increased respiratory tract infection rates in a small percentage of participants. A potential side effect associated with the inhibition of intracellular CTSS is the inhibition of MHC class II loading and subsequent decrease in adaptive immune responses. Inhibition of CTSS leads to decreased MHC class II expression on the surface of macrophages, monocytes and B cells and reduced expansion and activation of CD4+ T cells as well as suppressed B cell maturation to plasma cells [85, 142]. While this may have a beneficial effect in the treatment of autoimmune conditions such as lupus or rheumatoid arthritis, in conditions associated with infection, including pulmonary conditions such as CF, there is a risk that this could hinder the body’s ability to appropriately detect and respond to these infectious agents. This may have contributed to the increased respiratory tract infection rates observed in some participants and to the lack of trials in patients with pulmonary disease. However, we have previously demonstrated that E-64 administration to mice infected with respiratory syncytial virus reduced pulmonary symptoms and aided in viral clearance [228]. Therapies that specifically target extracellular CTSS, such as antibody or protein-based therapies, may be of greater benefit for these conditions. More generally, off-target effects are an issue with cathepsin inhibitors due to high homology between proteases in the family. This has been most notable in the development of CTSK inhibitors [229, 230]. However, X-ray crystallography has provided a more detailed understanding of the structure of the protease active sites allowing the development of more selective cathepsin inhibitors and mitigating the potential side effects of off-target inhibition [41].

## Conclusions

CTSS has extracellular and intracellular functions that can influence many physiological changes within the lungs and most importantly impact disease pathology. A number of studies suggest that CTSS has potential as a prognostic biomarker, for example, as a surrogate marker of lung disease progression. Targeting CTSS represents a potential therapeutic treatment for several pulmonary diseases and their comorbidities. Recent studies suggest that targeting CTSS may improve several symptoms of pulmonary diseases, such as mucus production, inflammation responses and lung function as observed in animal models of CF [73] and COPD [58, 99]. Improving our understanding of the CTSS degradome is essential to better understand the pathophysiological role of CTSS in health and disease. Importantly, the long-term impact of inhibiting CTSS extracellularly or intracellularly within the lung and systemically requires further investigation.

## Data Availability

Not applicable.

## Abbreviations

CTSS: Cathepsin S
CF: Cystic fibrosis
COPD: Chronic obstructive pulmonary disease
IPF: Idiopathic pulmonary fibrosis
NE: Neutrophil elastase
A1AT: α1-antitrypsin
SLPI: Secretory leukoprotease inhibitor
ECM: Extracellular matrix
MHC: Major histocompatibility complex
GAGs: Glycosaminoglycans
CLIP: Class II-associated leupeptin-induced peptide
AMP: s Antimicrobial proteins
BALF: Bronchoalveolar lavage fluid
PP2A: Protein phosphatase 2A
OVA: Ovalbumin
PAR: Protease-activated receptor
PAH: Pulmonary arterial hypertension
PASMC: Pulmonary arterial smooth muscle cells
MCT: Monocrotaline
ROS: Reactive oxygen species
PPAR: Peroxisome proliferator-activated receptor
ENaC: Epithelial sodium channel
STIM: Stromal interaction molecule
CIP2A: Cancerous inhibitor of PP2A
AAA: Abdominal aortic aneurysm
Ldlr: Low-density lipoprotein receptor
ApoE: Apolipoprotein E
ISRE: IFN-stimulated response element
Per: Period
HuR: Human antigen R
TTP: Tristetraprolin
GILT: γ-Interferon-inducible lysosomal thiol reductase
H_2_O_2_: Hydrogen peroxide
